# Circadian rapid eye movement sleep expression is associated with brain microstructural integrity in older adults

**DOI:** 10.1038/s42003-024-06415-y

**Published:** 2024-06-22

**Authors:** Michele Deantoni, Mathilde Reyt, Marine Dourte, Stella de Haan, Alexia Lesoinne, Gilles Vandewalle, Christophe Phillips, Christian Berthomier, Pierre Maquet, Vincenzo Muto, Grégory Hammad, Christina Schmidt, Marion Baillet

**Affiliations:** 1 GIGA-CRC Human Imaging, University of Liège, Liège, Belgium; 2 Psychology and Neuroscience of Cognition Research Unit (PsyNCog), Faculty of Psychology and Educational Sciences, University of Liège, Liège, Belgium; 3 GIGA-In Silico Medicine, University of Liège, Liège, Belgium; 4 Physip, Paris, France; 5 Department of Neurology, University Hospital of Liège, University of Liège, Liège, Belgium; 6 Human Chronobiology and Sleep, University of Surrey, Guildford, England

**Keywords:** Circadian regulation, REM sleep, Ageing

## Abstract

Rapid eye movement sleep (REMS) is increasingly suggested as a discriminant sleep state for subtle signs of age-related neurodegeneration. While REMS expression is under strong circadian control and circadian dysregulation increases with age, the association between brain aging and circadian REMS regulation has not yet been assessed. Here, we measure the circadian amplitude of REMS through a 40-h in-lab multiple nap protocol in controlled laboratory conditions, and brain microstructural integrity with quantitative multi-parameter mapping (MPM) imaging in 86 older individuals. We show that reduced circadian REMS amplitude is related to lower magnetization transfer saturation (MTsat), longitudinal relaxation rate (R1) and effective transverse relaxation rate (R2*) values in several white matter regions mostly located around the lateral ventricles, and with lower R1 values in grey matter clusters encompassing the hippocampus, parahippocampus, thalamus and hypothalamus. Our results further highlight the importance of considering circadian regulation for understanding the association between sleep and brain structure in older individuals.

## Abstract (web summary)

This study highlights the circadian regulation of rapid eye movement sleep as a neurophysiological correlate of subtle brain microstructural changes in key brain regions sensitive to aging and involved in sleep-wake regulation.

## Introduction

Human aging is characterized by large inter-individual differences in brain structure and function which is thought to be driven by multiple factors^1^. Among these factors, sleep has been increasingly suggested to play a protective role for brain aging^2–4^. Sleep-wake states are controlled by hypothalamic and brainstem nuclei that send projections to the thalamus and cortical areas to generate varying levels of arousal and associated vigilance states^5^. Wake-promoting nuclei appear particularly vulnerable to early disruption, which may, at least partially, underlie prominent changes in sleep in aging and neurodegenerative disease^6^. Age-related alterations in sleep classically include reduced sleep duration, advanced sleep timing, increased sleep fragmentation as well as reduced slow-wave sleep and comparatively smaller reductions in rapid eye movement sleep (REMS)^7^. Notably, even though REMS appears on average less affected during healthy aging, it has attracted increasing interest as a relevant marker to forecast cognitive decline and dementia risk in cognitively unimpaired older adults^8,9^. Within this context, grey matter atrophy, cortical thickness and white matter integrity have been associated with inter-individual differences in the overnight expression of REMS in older adults^10–12^.

REMS expression is strongly controlled by the circadian timing system^13,14^. In humans, the sleep-dependent increase of REMS has been shown to interact with its circadian modulation to result in highest REMS expression when sleep is scheduled to occur in the early morning hours^15^. Older age is associated with altered circadian sleep-wake promotion, including a less distinct circadian REMS generation^16,17^, which has been proposed to partially mediate age-related changes in sleep^17–19^.

While REMS may reflect a discriminant factor to track first signs of age-related brain alteration, the association between brain metrics and specific REMS regulation processes remains widely unexplored. Assessing a specific sleep regulation process rather than the expression of a sleep stage per se, which may be affected by different factors including environmental constraints and/or sleep-wake history, may be particularly relevant for understanding the association between sleep and brain structure during aging. In a recent exploratory study, we gathered first evidence that circadian REMS expression is associated with local cortical gyrification in healthy older adults^20^. Importantly, such changes or re-organisation at the macrostructural level are assumed to be preceded by microstructural tissue alteration^21^. Recent developments in quantitative magnetic resonance imaging (MRI) and biophysical modelling have improved the ability to study brain microstructure in vivo (referred to as in vivo histology using MRI), by providing MR features of tissue properties (e.g., relaxation times, magnetization transfer) that inform about underlying cellular and molecular processes^22^. Notably, previous studies revealed widespread age-related changes in quantitative multi-parameter mapping (MPM) parameters reflecting myelin, iron and water content, suggesting that these parameters may track subtle changes in processes of demyelination and iron deposition^23,24^.

Here, we explored the associations between age-sensitive MPM parameters and circadian REMS regulation measured through a multiple nap protocol. We hypothesized that reduced circadian REMS expression is related to altered brain microstructural integrity, particularly in regions shown to be sensitive to the aging process.

## Results

Demographic characteristics and sleep variables of participants are presented in Supplementary Table 1. The mean age ± standard deviation (SD) of the study sample was 68.9 ± 5.2 years, and 32 participants were females.

### Circadian modulation of sleep efficiency and REM sleep

**Fig. 1:**
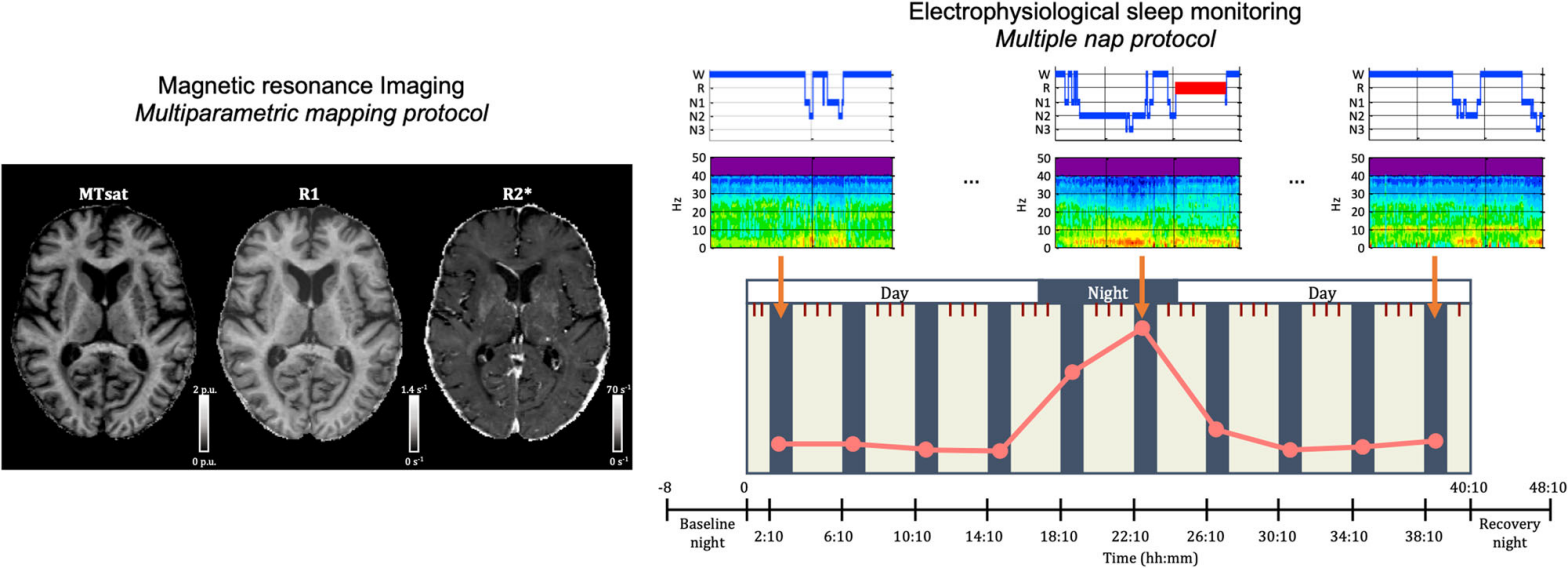
Schematic representation of the experimental protocol. During the first visit, a structural MRI session was performed on a 3 T MRI scanner. The multiparameter mapping protocol was acquired to compute MTsat, R1 and R2* maps. On a separate visit, participants underwent a 40-h in-lab multiple nap protocol encompassing 10 short sleep-wake cycles, each consisting of 160 min of wakefulness (dark blue) alternating with 80 min of sleep opportunities (light beige). The multiple nap protocol was preceded and followed by 8 h of sleep opportunity (baseline and recovery night, respectively). Sleep stages were extracted based on spectral composition of electroencephalographic signals acquired during sleep opportunities. Light levels (4.5–5 lux during wakefulness and <1 lux during sleep), temperature (~19 °C), caloric intake and body posture (semi-recumbent position during scheduled wakefulness and recumbent during sleep opportunities) were controlled. The timing of nap sessions was aligned with the individual’s scheduled wake-up time from the baseline night (elapsed time expressed in hours). The time-of-day axis indicates corresponding clock time for an individual waking up at 07:00 and going to sleep at 23:00. Salivary melatonin (brown short lines) was collected over the 40-h multiple nap protocol, with an average sampling rate of 80 min starting 50 min after wake up. The pink curve represents a schematic representation of circadian REMS modulation that can be extracted from the protocol.

**Fig. 2:**
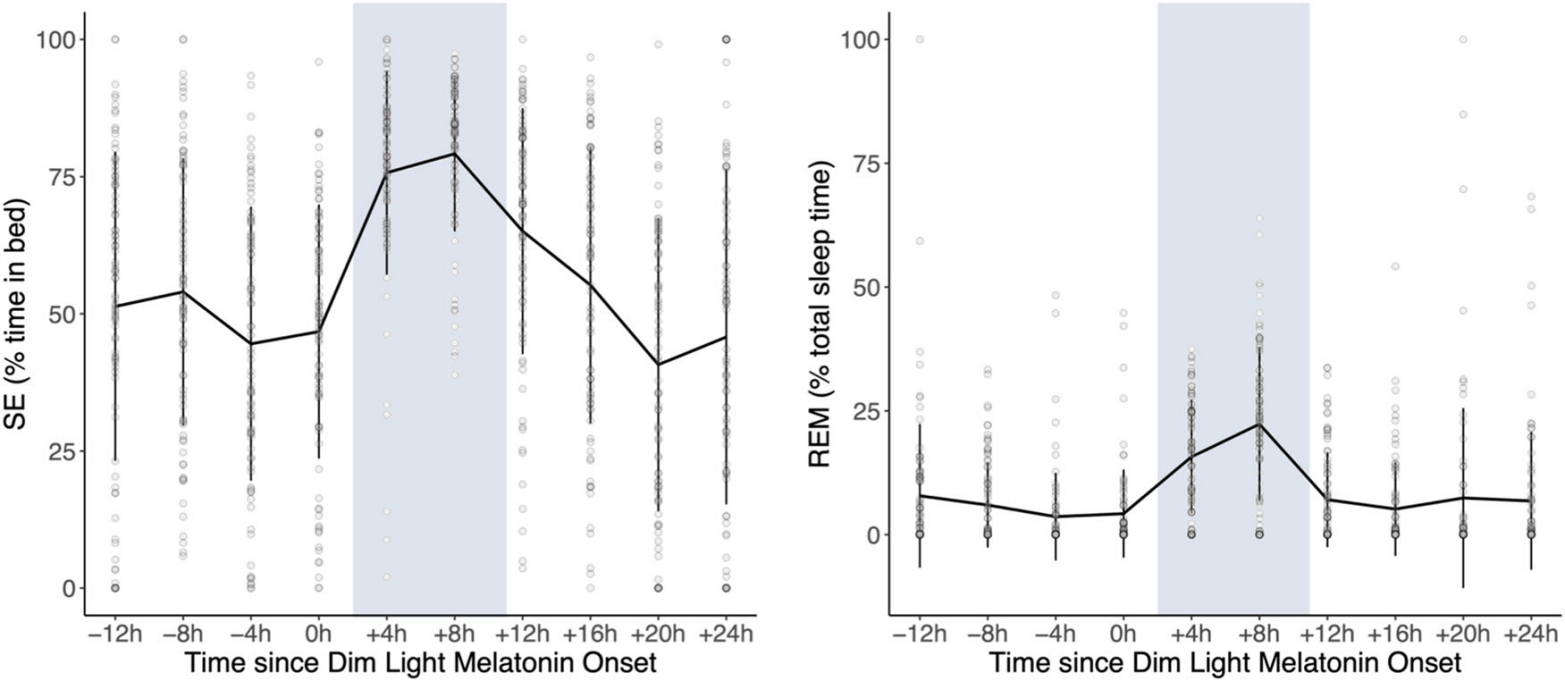
Time course of SE (expressed as % of sleep opportunity) and REM sleep (expressed as % of total sleep time) across the nap opportunities over the 40-h multiple nap protocol (mean ± standard deviation, *N* = 86). The blue area represents the participants’ typical nighttime sleep period. The timing of each nap is shown relative to the distance from DLMOn.

Participants underwent a 40-h multiple nap protocol in controlled laboratory conditions allowing for the measurement of sleep and REMS duration on each nap opportunity at different circadian phases (Fig. 1). As depicted in Fig. 2, both SE and REM% exhibited a circadian modulation over the protocol. More specifically, compared to nighttime nap opportunities, SE was significantly reduced when sleep opportunities were scheduled during the biological day, and particularly around the end of the day (*p* < 0.001 for time since dim light melatonin onset [DLMOn] −12 h, −4 h, 0, +12 h, +20 h, +24 h, β = −0.29, β = −0.43, β = −0.34, β = −0.39, β = −0.70, β = −0.38, respectively). Likewise, REM% was significantly higher during nighttime naps compared to daytime naps (*p* < 0.001 for time since DLMOn −4 h, β = −0.41; *p* < 0.05 for time since DLMOn 0 h, +16 h, +20 h, +24 h, β = −0.29, β = −0.25, β = −0.25, β = −0.22, respectively), and during the second nighttime nap compared to the first nighttime nap (time since DLMOn +8 h > +4 h, *p* < 0.05, β = −0.29). Finally, we observed a positive association between circadian REMS amplitude and REM% measured during the baseline night, after accounting for age, sex, and SE (t(4, 81) = 3.32, *p* = 0.001).

### Association between age and brain microstructure

**Fig. 3:**
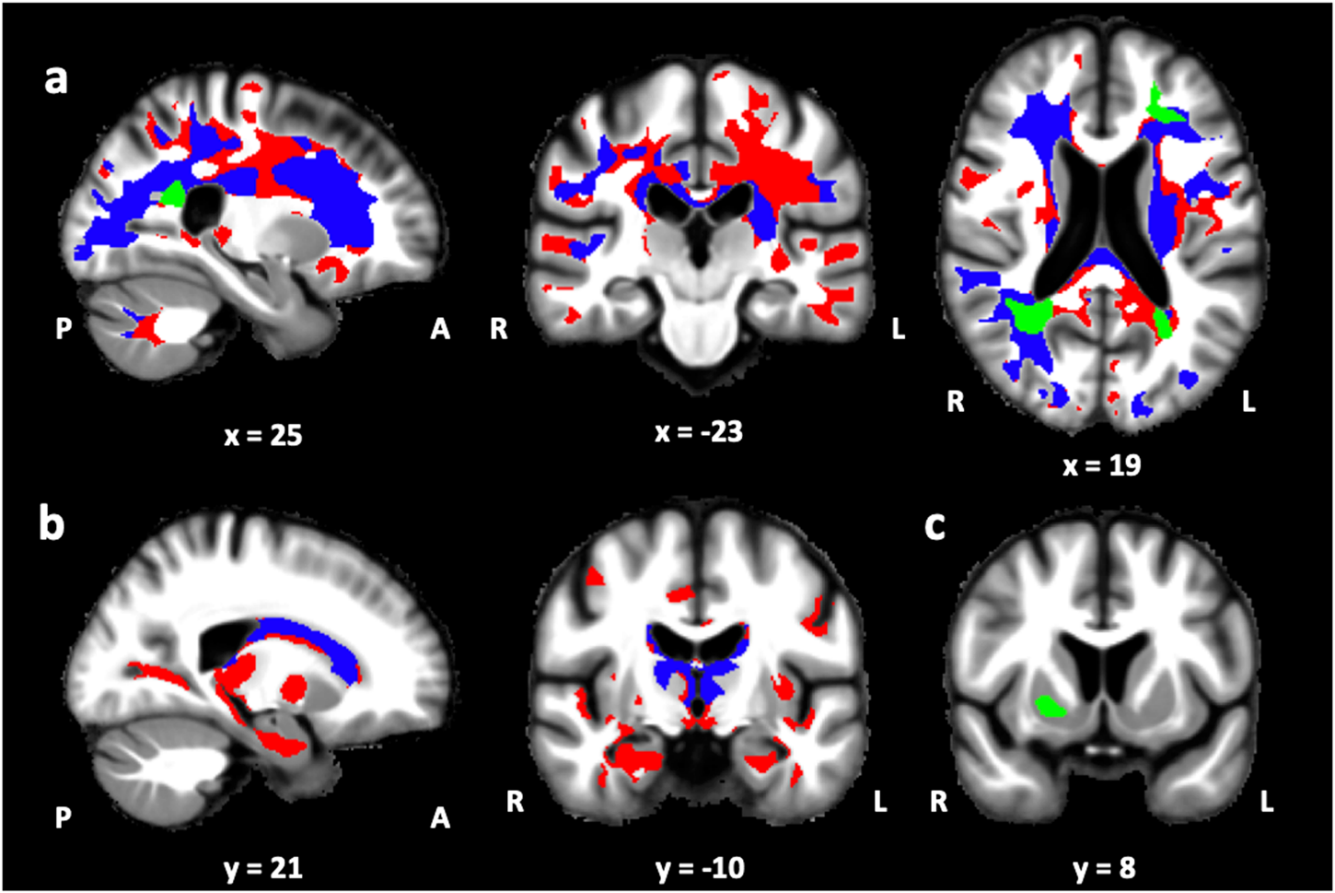
Association between age and white or grey matter microstructural integrity (*N* = 86). **a** Negative association between age and white matter MTsat (red), R1 (blue), and R2* (green) values, (**b**) Negative association between age and grey matter MTsat (red) and R1 (blue) values, (**c**) Positive association between age and grey matter R2* values (green). Results are displayed at *p* < 0.05 FWE corrected and overlaid on the average normalized MTsat map of the population.

On a separate visit, participants underwent an MRI scanning session. Grey and white matter microstructural tissue properties were derived from magnetization transfer saturation (MTsat), longitudinal relaxation rate (R1), and effective transverse relaxation rate (R2*) maps obtained with a quantitative MPM protocol. As expected, MPM parameters were significantly impacted by age in grey and white matter (Fig. 3 and Supplementary Tables 2, 3). We observed a widespread decrease in white matter MTsat values with older age and, to a lesser extent, in R1 values mainly over frontal and parietal areas as well as more locally decreased R2* values in regions including parts of the corona radiata, body of the corpus callosum, thalamic radiation and fornix. Similarly, older age was related to decreased MTsat and R1 values in several cortical and subcortical grey matter regions, including the thalamus, the medial temporal lobe, the caudate, the pallidum, and the ventral diencephalic region (mostly hypothalamus). Finally, we observed a positive association between R2* values and age in the putamen.

### Association between circadian REM sleep expression and brain microstructure

**Fig. 4:**
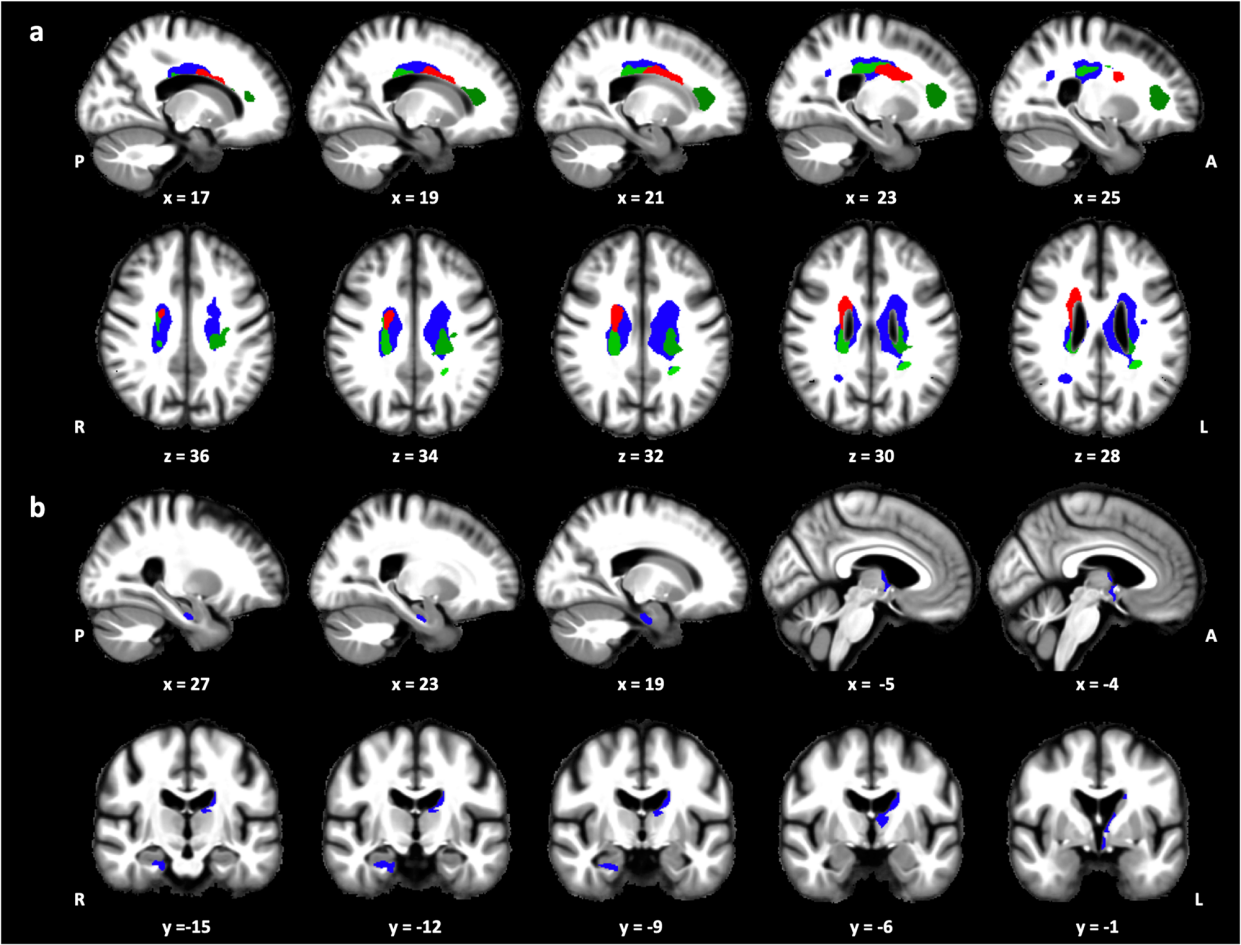
Association between circadian amplitude of REM sleep expression and white or grey matter microstructural integrity (*N* = 86). **a** Positive association between circadian REM sleep amplitude and white matter MTsat (red), R1 (blue), and R2* (green) values. **b** Positive association between circadian REM sleep amplitude and grey matter R1 values (blue). Results are displayed at *p* < 0.05 FWE corrected and overlaid on the average normalized MTsat map of the population.

The circadian modulation of REMS was computed by fitting a Gaussian function on REM% derived from each nap opportunity. Circadian REMS amplitude was then defined as the height of the fitted gaussian function (see Supplementary Fig. 1 for a distribution of circadian REMS amplitude values). After adjusting for demographics and additional variables related to sleep architecture and its circadian modulation, including circadian SE amplitude and REM% measured during the baseline night, whole-brain voxel-wise quantification analyses revealed a positive association between circadian REMS amplitude and MTsat, R1, and R2* values in several white matter clusters mainly located around the lateral ventricles (Fig. 4a, Supplementary Table 4). In grey matter, we observed a significant positive association between circadian REMS amplitude and R1 values in clusters encompassing the ventral diencephalic region, mostly including the hypothalamus, the anterior part of the thalamus, the hippocampus and the parahippocampal gyrus (Fig. 4b, Supplementary Table 5). Furthermore, no significant results were observed when testing the relationship with grey matter volume, nor when assessing negative associations between circadian REMS amplitude and grey/white matter MPM values (i.e., inverse contrast). Finally, we did not find significant associations between circadian SE amplitude and grey/white matter MPM values.

## Discussion

Recently, REMS has gained increasing interest as a relevant and early sleep marker for cognitive decline, dementia risk, and age-related brain changes^8,9,11,12^. Considering that REMS expression is strongly controlled by the circadian timing system, the aim of this study was to investigate the association between circadian REMS regulation and brain microstructural integrity in a group of healthy older adults. We observed that reduced circadian REMS amplitude is associated with lower nighttime REM% and altered microstructural integrity in brain regions highly sensitive to the aging process and involved in sleep-wake regulation. Overall, these findings extend those from previous studies by highlighting the daily dynamics of REMS as a neurobiological correlate of age-related changes in brain tissue microstructure. Considering that circadian REMS regulation contributes to consolidated overnight REMS expression, this study supports that the association between REMS and brain integrity may be partially pinned down to altered circadian REMS regulation and further emphasizes the relevance of investigating sleep regulation processes to better understand the association between specific sleep phenotypes and brain structure during aging.

Quantitative MRI provides useful information for tracking subtle tissue changes that occur during aging^22^. Previous quantitative MPM studies reported widespread age-related reduction in MT/MTsat and R1 values over white matter, and an increase in R2* values in subcortical regions^23,24^, a pattern that is similarly observed in our study. Since myelin is the main component of white matter^25^, the decrease in white matter MTsat, R1 and R2* values observed in our study may indicate an age-related demyelination process. Likewise, postmortem studies revealed that MT values are related to myelin content in demyelinating diseases^26^ and R2* to iron concentration^27^. In cortical grey matter, however, MTsat and R1 are more likely to be associated with cell membrane proteins and phospholipids. At the cellular level, previous studies reported that MTsat and R1 are related to cytoarchitecture in the cerebral cortex, including neuronal and non-neuronal cells such as microglia and endothelial cells^28,29^. The decrease in MTsat and R1 values observed in grey matter may therefore indicate an age-related reduction in non-neuronal cell and/or neurite density. Finally, an increase in R2* is commonly reported in basal ganglia and is thought to reflect iron accumulation over the lifespan^23,30^.

Our analysis revealed that reduced circadian REMS amplitude, but not overall circadian sleep amplitude, was related to decreased R1 values in grey matter as well as decreased MTsat, R1 and R2* values in white matter. The concomitant decrease in MTsat, R1 and R2* values in white matter bundles mainly located around the ventricles suggests a loss of white matter microstructural integrity, including demyelination processes. The periventricular white matter zone is commonly observed to be affected during aging through the presence of white matter hyperintensities^31^. These white matter hyperintensities are related to white matter tissue damage, including demyelination, axonal loss and gliosis, and are linked to clinical trajectories in aging^32^. White matter hyperintensities are driven by small vessel disease, which involves damage to ependymal cells leading to cerebrospinal fluid infiltration into perivascular brain tissues^31^. Notably, circadian rhythms and REMS have both been shown to regulate blood brain barrier properties^33,34^, suggesting a potential underlying mechanism linking circadian REMS regulation to white matter microstructure in aging. In a broader context, our findings are also consistent with previous actigraphic studies showing that an altered 24-hour rest-activity rhythmicity is associated with reduced white matter integrity and higher white matter hyperintensity volume^35–37^.

In contrast to white matter, R1 was the only parameter associated with circadian REMS amplitude in grey matter. This observation could be partially explained by the relative non-specificity of R1, which rather represents a composite measure of the different effects measured by the other two parameters. Indeed, R1 has been shown to be primarily impacted by macromolecular content (e.g. myelin, cell membrane lipids and proteins) but also, to a lesser extent, by iron content^38^.

We observed that reduced circadian REMS amplitude was related to decreased R1 values in the hippocampus and the parahippocampus suggesting an altered macromolecular content in these regions, most likely associated with membrane lipids and proteins and reflecting changes in non-neuronal cell and/or neurite density. Notably, the hippocampus is crucially involved in theta wave generation that is typically occurring during REMS and that has been suggested to subserve sleep-dependent memory consolidation^39^. Furthermore, medial temporal lobe regions are commonly affected during aging, with the hippocampus and the parahippocampus volumes declining after midlife^40^. Medial temporal lobe atrophy has been related to age-related cognitive decline and more particularly episodic memory performance^41^, and represents a common MRI marker to track neurodegeneration in clinical routines^42^. Finally, actigraphy-derived 24-h rest-activity rhythms fragmentation has been previously associated with medial temporal lobe atrophy in older adults^43^.

We also observed a positive association between circadian REMS amplitude and grey matter R1 values in diencephalic brain regions, mainly covering the anterior thalamus but also large parts of the hypothalamus. These structures are critically involved in the daily modulation of arousal and sleep-wake states^5^. Interestingly, while we observed that low circadian REMS amplitude was related to low nighttime REM sleep propensity, the association between brain tissue microstructural indices and circadian REMS amplitude in these regions was significant after accounting for nighttime sleep and REM%. We speculate that altered circadian REMS regulation may therefore result from early age-related degeneration in wake-promoting nuclei^6^ receiving inputs from the central circadian clock located in the suprachiasmatic nucleus (SCN), such as orexinergic neurons of the posterior hypothalamus as previously suggested^14,44^. In addition, the SCN, which mainly provides temporal organization to the sleep-wake cycle^45^, has also been suggested to promote REMS at specific times of the day^14,15^. While our data do not allow to pinpoint the association between circadian REMS expression and microstructural integrity to specific hypothalamic nuclei, they nevertheless suggest that changes in the daily dynamics of REMS are linked to microstructural changes in diencephalic brain regions, that are at the core of sleep and wakefulness regulation or consolidation over the 24-h cycle. Within this context, the increased occurrence of habitual daytime napping in older adults may reflect a visible manifestation of altered sleep-wake cycle consolidation. In our sample, 65% of the participants reported to regularly nap during the day and we recently observed that this phenotype is associated with altered dynamics of REMS generation over the 24-h cycle^46^.

Potential limitations should also be discussed in the context of the present study. First, previous studies reported that R2* maps are influenced by fiber orientation, which may affect the interpretation of our results^47^. However, the results observed with R2* maps were consistent with those observed for MTsat and R1 maps, supporting the interpretation toward an age-related demyelination process. Then, while we observed a significant association between circadian REMS regulation and microstructural integrity within the hypothalamus, our whole brain voxel-wise analysis does not allow to identify an association with specific hypothalamic subregions. Similarly, other wake-promoting nuclei located in the brainstem (e.g., locus coeruleus, raphe nucleus) or in the basal forebrain may constitute potential neuronal substrates of circadian REMS regulation because of their involvement in REMS physiology and/or their connections with the SCN^5^. However, the nature of the current whole-brain analysis and the lack of dedicated sequences to delineate these nuclei limit the investigation of their structural integrity in vivo. Future regions of interest studies using for example recently developed algorithms of hypothalamic subfields parcellation^48^ or high-resolution MRI are therefore warranted to improve the regional specificity of our findings and to elucidate the contribution of additional sleep-wake nuclei to circadian REMS regulation. In addition, while we only considered the circadian modulation of REMS expression, REMS characteristics (e.g. spectral composition) and their circadian modulation should be investigated to determine their association with microstructural brain tissue changes. Finally, additional studies are needed to investigate whether circadian REMS changes are accompanied by age-related cognitive decline and brain markers usually explored in the context of neurodegenerative diseases (e.g., amyloid-β and tau deposition).

The aim of this study was to investigate whether circadian REMS regulation is associated with brain tissue microstructure in older individuals. Our findings highlight the circadian regulation of REMS as a neurophysiological correlate of subtle brain microstructural changes, including demyelination and decreased non-neuronal cell and/or neurite density that occur in key brain regions sensitive to aging and involved in regulating sleep and wakefulness, and stress the importance of considering circadian regulation for understanding the association between sleep and neurodegeneration in aging.

## Methods

### Participants

Eighty-six healthy retired older participants aged between 59–82 years were recruited. Inclusion criteria have been described elsewhere^49^. Briefly, participants were screened for psychoactive drug impacting the central nervous system, major sleep disorders (mean apnea/hypopnea index [events/hour] ± SD = 5.8 ± 4.8; mean periodic limb movement events/hour ± SD = 3.7 ± 8.2), recent psychiatric disorders (moderate/severe depression, Beck Depression Inventory score > 19; severe anxiety, Beck Anxiety score > 30), brain trauma, diabetes and body mass index ≤ 18 kg/m² and ≥ 30 kg/m², smoking, excessive alcohol ( > 14 units/week) or caffeine ( > 4 cups/day) and drug consumption, and cognitive status (Mini Mental State Examination score < 26, Mattis Dementia Rating Scale score < 130). In the context of a broader study, participants were further screened with regards to their napping habits, which was subjectively assessed through a questionnaire. Prospective nap recruitment can be considered as a tool to enhance inter-individual variation in sleep-wake phenotypes and more particularly in the temporal dynamics of sleep-wake regulation^46^. Out of the current sample, 56 (65%) subjectively reported to regularly nap ( ≥ 3 naps/week, for at least one-year preceding study entrance) while 30 (35%) declared not to nap. Recruitment was performed through access to a GDPR-compliant database in the laboratory and via study advertisement in newspapers. The study was approved by the local Ethics Committee of the University Hospital and of the Faculty of Psychology, Logopedics and Educational Sciences at the University of Liège (Belgium) and was conducted in accordance with the Declaration of Helsinki. All ethical regulations relevant to human research participants were followed. Participants provided written informed consent and received financial compensation.

### Experimental protocol

After completing a screening night of polysomnography, participants underwent an MRI session allowing for the assessment of brain structural integrity (Fig. 1). During the week prior to laboratory entrance, participants were asked to follow a pre-determined and individually-adapted sleep-wake schedule, centered around an 8-hour nighttime sleep opportunity with a maximal deviation of 30 min to ensure sufficient sleep and stable circadian entrainment to the selected regime. Sleep schedules (i.e. sleep and wake-up times from baseline night and subsequent nap timing) were individually adapted to the participants’ preferred sleep-wake times, centered on an 8-h sleep opportunity. The alignment of protocol timing to individually scheduled wake-up times, rather than absolute clock time, has the advantage to allow for inter-subject comparisons across nap sessions, considering both time since awake and circadian time. Compliance with the schedule was verified by actigraphic recordings. Consumption of alcohol and energy drinks was prohibited during this week to prevent withdrawal effects during the in-lab protocol.

After entering the laboratory, participants completed a 40-hour multiple nap protocol (Fig. 1) encompassing 10 short sleep-wake cycles of 80 min of sleep opportunity (i.e., a nap) alternating with 160 min of scheduled wakefulness. The multiple nap protocol was preceded and followed by an 8-hour baseline and recovery night. The first cycle started 130 min after scheduled wake-up time from the baseline night. The duration of wakefulness in the last cycle was restricted to 40 min such that the recovery night started at habitual sleep time. During the entire in-lab protocol, controlled laboratory conditions were applied including lighting (4.5–5 lux during wakefulness and < 1 lux during scheduled sleep opportunities, see also^49^ for further specifications about light settings), ambient temperature of approximately 19°C, semi-recumbent body posture during wake periods and recumbent during sleep opportunities, and isocaloric food intake (individually standardized meals every 4 h). Participants were not allowed to stand up, except for regularly scheduled bathroom visits, and they did not have any indications of time of day. Social interaction was restricted to communications with study staff. Salivary melatonin was collected at regular intervals ( ~ 1.25 h) throughout the 40-hour protocol for circadian phase assessment.

### Circadian phase assessment: melatonin

Before collection of saliva samples, participants were not allowed to eat for 30 min, nor to drink water or change their posture for 15 min. Salivary melatonin analysis was carried out at the Department of Clinical Chemistry, University of Liège, Belgium. Saliva samples were kept at −80 degrees Celsius until they were analyzed using liquid chromatography coupled to a tandem mass spectrometer. 500 μL of saliva samples were extracted using a liquid-liquid extraction method before being separated using a Nexera X2 UPLC (Shimadzu, Kyoto, Japan) on a C18 column. A QTrap6500 mass spectrometer (Triple Quadrupole and Linear Trap Analyzers, Sciex, CA, USA) was used to analyze and quantify the extracts^50^. A total of 30 samples were acquired per participant over the 40-h multiple nap protocol ( ~ 3 samples in between the scheduled nap sessions, see Fig. 1). A skewed baseline cosine function was used to determine secretion profiles^51^. The timing of dim light melatonin onset (DLMOn) and dim light melatonin offset (DLMOff) were extracted to assess circadian phase. DLMOn was defined as the point when melatonin levels reached 25% of the fitted peak-to-baseline amplitude of individual data.

### Sleep data acquisition and analysis

Sleep was measured using seven electroencephalographic (EEG) channels (Fz, C3, Cz, C4, Pz, Oz, O2), two bipolar electrooculograms and two bipolar submental electromyograms. Signals were recorded with Ag/AgCl ring electrodes and N7000 amplifiers (EMBLA, Natus Medical Incorporated, Planegg, Germany). The sampling rate was set at 500 Hz and signals were filtered online by applying a notch filter (50 Hz). Out of the 860 nap opportunities (10 for each participant), 2 naps were missing due to technical problems during the acquisition phase.

Sleep stages were scored automatically in 30-second epochs according to the American Academy of Sleep Medicine criteria (AASM^52^) using the ASEEGA sleep scoring algorithm (ASEEGA, PHYSIP, Paris, France), based on spectral composition of electroencephalographic signals acquired during sleep opportunities. We previously reported the use of this automatic algorithm to score sleep and REMS in the context of napping in young and older individuals^20^. In addition, the consistency between the hypnograms and the spectral plots of the ~860 nap recordings was visually inspected by a trained sleep scorer to ensure that the algorithm did not overlook any major episodes of REMS. During each sleep opportunity, sleep efficiency (SE, sum of time spent in sleep stages 1, 2, 3 and REMS divided by total sleep opportunity) and REMS (REM%, expressed as a percentage over total sleep time) were computed. Individual values were interpolated (3^rd^ order b-spline) at the following theoretical circadian times over the protocol: −12 h, −8 h, −4 h, 0 h, 4 h, 8 h, 12 h, 16 h, 20 h, 24 h from DLMOn. To measure the circadian modulation of REM%, our main variable of interest, and SE, their profile over time were fitted with the convolution of a first-order polynomial and a Gaussian function. The minimum of the fit was restricted to occur within a 4 h window surrounding the DLMOn. Circadian REM% or SE amplitude was defined as the height of the fitted gaussian function, while the accumulation of REM% or SE during the protocol was estimated by the slope of the first-order polynomial component. Finally, mean REM% and SE over the naps as well as REM% and SE during the baseline night were also computed for each participant.

### MRI data acquisition and processing

MRI scans were conducted using a 3 T scanner (MAGNETOM Prisma, Siemens) equipped with a 64-channel head coil. The MPM protocol was composed of three co-localized multi-echo 3D FLASH scans^53^ acquired with predominant T1, proton density (PD) and magnetization transfer (MT) weighted with TR/FA = 24.5 ms/21° for T1 weighted (T1w), TR/FA = 24.5 ms/6° for MT weighted (MTw) and PD weighted (PDw), and with a voxel size of 1 × 1 × 1 mm^3^. One radio frequency (RF) transmit field map and one B0 field map were also acquired to correct for B1 field bias^54^ and image distortion^53^, respectively. To provide MTw, an off-resonance Gaussian RF pulse of 5 ms duration, 220° nominal flip angle, 2 kHz frequency offset was performed prior to the excitation. Two unaccelerated, low resolution (8 mm isotropic) volumes were acquired prior to the FLASH scans (TR = 6 ms, TE = 2.20 ms, FA = 6°), using respectively the 64-channel coil for signal reception and the body coil for signal reception to correct for the relative receive field sensitivity of the array coil, which is position-specific^55^. B1 maps were extracted from the spin echo (SE) and stimulated echo (STE) signals of a fast 3D multishot echo-planar imaging (EPI) sequence^56^. The multishot EPI data were acquired with the following parameters: matrix size = 64 × 48 × 48, FOV = 256 × 192 × 192 mm³, TE(SE)/TE(STE)/TM/TR = 25.5/51.0/40.0/500 ms, excitation nominal FA = 90°. The respective flip angles for the SE/STE pulses varied between 130/65° and 280/140° in steps of 15/7.5°.

The multi-parametric echo (PDw, T1w, and MTw) images were auto-reoriented against an MNI template and MTsat, PD, R1 and R2* maps were created using the hMRI toolbox (v0.2.2, http://hmri.info)^57^ within the SPM12 environment (Statistical Parametric Mapping, Wellcome Centre for Human Neuroimaging, London, UK, http://www.fil.ion.ucl.ac.uk/spm, University College London, revision 12.6). Contrary to the commonly used MT ratio (percentage reduction in steady-state signal), the MTsat map explicitly accounts for spatially varying T1 relaxation time and flip angles^58^. Grey matter and white matter probability maps, derived from the segmentation of the MTsat maps, were used to create a study-specific DARTEL template. MTsat, R1 and R2* maps were then non-linearly and linearly warped to the MNI space using the subject-specific diffeomorphic estimates from the DARTEL procedure and an affine transformation, respectively. Grey matter probability maps were similarly processed into the MNI space, with the addition of modulation by the Jacobian determinant of the warps to preserve the total amount of grey matter. MPM maps were finally smoothed with a kernel of 6 mm using a tissue-specific weighting which accounts for partial volume distribution of white and grey matter tissue in each voxel^24^ and used for voxel-based quantification (VBQ) analysis. MTsat, R2* and R1 provides crucial information about the local microstructural environment, with MTsat interpreted as a marker of macromolecular content, including myelin, R2* as an indicator of iron content, and R1 as a marker for macromolecular content with some sensitivity to iron^23^. Finally, modulated grey matter maps were smoothed with a kernel of 8 mm and used for additional voxel-based morphometry (VBM) analysis.

### Statistics and reproducibility

Generalized linear mixed models were performed to assess the effect of nap session (within-subject factors) on REMS (REM%) and SE values aligned to DLMOn using the package *glmmTMB* (v1.1.5) of the R software (v4.2.2). Subjects were defined as random effects and sessions as categorical fixed effects. User-defined contrasts were used to compare REM% and SE measured during each nap (i.e., biological day) to their mean (REM% and SE, respectively) computed over the two-night sessions (i.e., biological night, time since DLMOn +4 h, +8 h). Separate models were performed for REM% and SE. To further investigate whether circadian regulation of REMS is associated with its overnight expression, a linear regression model was conducted between circadian REM amplitude and REM% during the baseline night adjusted for age, sex, and SE. Statistical significance was set at two-tailed *p* < 0.05.

Whole-brain VBQ analyses were performed in the framework of SPM12. For each MPM map, a multiple linear regression model was computed to test for the relationship between the circadian amplitude of REMS (fitted amplitude of the Gaussian curve), as the main variable of interest, and MTsat, R1 and R2* values over grey/white matter. Statistical models were adjusted for age, sex, education, and for additional covariates related to sleep architecture and its circadian modulation, including circadian amplitude of SE, mean REM% and SE computed over the protocol, accumulation of REMS and sleep need (slope of the fitted first-order polynomial curve), and REM% and SE measured during the baseline night to account for potential differences in sleep and REMS expression during the previous night. Finally, to investigate whether changes in grey matter tissue microstructure could be related to decreased grey matter volume, an additional VBM analysis was performed on grey matter maps. Grey and white matter tissue masks were computed by averaging smoothed Jacobian-modulated tissue probability maps, derived from the segmentation of MTsat maps, for all participants and by assigning voxels to the tissue class for which the probability was maximal. This step ensured that each voxel was analyzed in only one tissue class. In addition, voxels for which the probability to belong to grey matter and white matter did not exceed 20% were removed to exclude non-brain tissue^23^. Inferences were performed using whole-brain family-wise error (FWE) corrected *p*-values with a threshold set at *p* < 0.05, at the cluster level (cluster forming threshold at *p* < 0.0001 uncorrected at the voxel level to favor parsimony in the construction of the model). For anatomical labelling of grey matter, the Neuromorphometrics atlas was used (http://Neuromorphometrics.com/).

### Reporting summary

Further information on research design is available in the Nature Portfolio Reporting Summary linked to this article.

### Supplementary information

Supplementary Materials

Description of Additional Supplementary Materials

Supplementary Data 1

Reporting Summary

## Data Availability

All data supporting this study is openly available at the following repository: 10.5281/zenodo.8434929. The source data behind the graphs can be found in Supplementary Data 1.

## Code availability

All codes supporting this study are openly available at the following repository: 10.5281/zenodo.8434929.
