# Supplementary material for: Circadian rapid eye movement sleep expression is associated with brain microstructural integrity in older adults
Source: Commun Biol. 2024 Jun 22;7:758. doi: 10.1038/s42003-024-06415-y (PMC11193799; doi:10.1038/s42003-024-06415-y)
Supplement: Supplementary file 1 — Supplementary Materials [file 42003_2024_6415_MOESM1_ESM.pdf]

Title: Circadian rapid eye movement sleep expression is associated with brain microstructural integrity in older adults

Authors: Michele Deantoni[1], Mathilde Reyt[1,2], Marine Dourte[1,2], Stella de Haan[1], Alexia Lesoinne[1], Gilles Vandewalle[1], Christophe Phillips[1,3], Christian Berthomier[4], Pierre Maquet[1,5], Vincenzo Muto[1], Grégory Hammad[1,6], Christina Schmidt[1,2]*, Marion Baillet[1]*

Affiliations

1. GIGA-CRC Human Imaging, University of Liège, Liège, Belgium
2. Psychology and Neuroscience of Cognition Research Unit (PsyNCog), Faculty of Psychology and Educational Sciences, University of Liège, Liège, Belgium
3. GIGA-In Silico Medicine, University of Liège, Liège, Belgium
4. Physip, Paris, France
5. Department of Neurology, University Hospital of Liège, University of Liège, Liège, Belgium
6. Human Chronobiology and Sleep, University of Surrey, Guildford, England

* These authors jointly supervised this work

Supplementary materials

Supplementary Table 1: Participants characteristics.

Supplementary Table 2: Statistical VBQ outputs of the association between age and MTsat, R1 or R2* grey matter values.

Supplementary Table 3: Statistical VBQ outputs of the association between age and MTsat, R1 or R2* white matter values.

Supplementary Figure 1: Distribution of circadian REM sleep amplitude values.

Supplementary Table 4: Statistical VBQ outputs of the association between circadian REMS amplitude and MTsat, R1 or R2* white matter values.

Supplementary Table 5: Statistical VBQ outputs of the association between circadian REMS amplitude and MTsat, R1 or R2* grey matter values.

Supplementary Table 1: Participants characteristics

**Variables**	**Mean ± Sd or no. (%) [min - max]**
**N**	86
Demographic and clinic	
Age (years)	68.9 ± 5.2 [59 - 82]
Sex	
Female	32 (37%)
Male	54 (63%)
Education (years)	14.5 ± 3.2 [9 - 25]
Ethnicity	Caucasian
Mini-mental state examination	29.3 ± 0.8 [27 - 30]
Sleep measures during the baseline night	
Sleep duration (minutes)	384.6 ± 40.6 [279 - 462]
Sleep efficiency, %	80.2 ± 8.4 [58 - 95]
Rapid eye movement sleep duration, %	23.2 ± 6.8 [6 - 40]

Sd: standard deviation

Supplementary Table 2: Statistical VBQ outputs of the association between age and MTsat, R1 or R2* grey matter values.

	Cluster p(FWE-corr)	Cluster size (n voxels)	Peak coordinates (mm) x	y	z	Main brain regions
**MTsat (negative association)**	<0.001	51710	-7<br>32<br>31	-1<br>-32<br>-17	3<br>2<br>-11	Left/Right Thalamus, Left/Right Hippocampus, Left/Right Calcarine Cortex, Left/Right Caudate, Left/Right Cuneus, Left/Right Pallidum, Brainstem, Left/Right Planum Polare, Left/Right Ventral Diencephalon, Left/Right Transverse Temporal Gyrus, Right Entorhinal Cortex, Right Parahippocampal Gyrus
	0.045	415	-16	-26	42	Left Medial Precentral Gyrus, Left Middle Cingulate gyrus, Left Posterior Cingulate Gyrus, Left Supplementary Motor cortex
	0.001	1162	-5<br>0<br>-7	-17<br>-29<br>-40	30<br>26<br>24	Left/Right Middle Cingulate Gyrus, Left/Right posterior Cingulate Gyrus
	0.008	723	-44<br>-51<br>-46	-17<br>-13<br>-12	38<br>39<br>31	Left Postcentral Gyrus, Left Precentral Gyrus
	0.004	858	-1<br>-3<br>-6	-32<br>-25<br>-43	71<br>75<br>76	Left Medial Precentral Gyrus, Left Medial Postcentral Gyrus, Left Precentral Gyrus, Left Postcentral Gyrus
	0.006	774	-26<br>-34	-91<br>-88	7<br>7	Left Inferior Occipital Gyrus, Left Middle Occipital Gyrus, Left Superior Occipital Gyrus, Left Occipital Pole
	0.006	761	-22<br>-31	7<br>-4	57<br>51	Left Middle Frontal Gyrus, Left Superior Frontal Gyrus
	0.03	478	4<br>15	-11<br>-20	45<br>46	Right Middle Cingulate Gyrus, Right Medial Precentral Gyrus, Right Supplementary Motor Cortex
	0.007	737	66<br>51<br>57	-29<br>-23<br>-27	0<br>-9<br>-1	Right Superior Temporal Gyrus, Right Middle Temporal Gyrus
	0.01	671	13<br>4<br>4	16<br>15<br>1	39<br>40<br>50	Right Supplementary Motor Cortex, Right Middle Cingulate Gyrus
	<0.001	1573	28<br>34<br>39	-67<br>-71<br>-60	-9<br>-23<br>-25	Right Cerebellum Exterior, Right Occipital Fusiform Gyrus, Right Fusiform Gyrus
	0.015	597	48<br>54	-67<br>-66	1<br>7	Right Inferior Occipital Gyrus, Right Middle Temporal Gyrus
	0.046	410	-41<br>-39	-13<br>-5	-28<br>-38	Left Fusiform Gyrus, Left Inferior Temporal Gyrus
	0.044	417	14<br>4	33<br>35	25<br>26	Right Anterior Cingulate Gyrus, Right Medial Superior Frontal Gyrus
	0.036	451	36<br>45<br>40	-15<br>-10<br>-17	46<br>52<br>53	Right Precentral Gyrus, Right Postcentral Gyrus
**R1 (negative association)**	<0.001	2033	10<br>3<br>-7	-82<br>-89<br>-80	6<br>8<br>10	Right/Left Calcarine cortex, Right/Left Cuneus, Right/Left Lingual Gyrus
	<0.001	6762	11<br>21<br>19	-2<br>18<br>1	12<br>16<br>28	Right/Left Thalamus, Right/Left Caudate, Right Ventral Diencephalon
**R2* (positive association)**	0.003	1305	24<br>13	6<br>19	-5<br>-4	Right Putamen, Right Caudate, Right Accumbens, Right Pallidum

Supplementary Table 3: Statistical VBQ outputs of the association between age and MTsat, R1 or R2* white matter values.

	Cluster p(FWE-corr)	Cluster size (n voxels)	Peak coordinates (mm) x	y	z
**MTsat (negative association)**	<0.001	13373	52	-36	-20
			65	-21	4
			63	-9	0
	<0.001	163847	-22	4	48
			-35	6	55
			-52	-4	37
	0.022	1261	-39	-63	-37
			-34	-57	-46
			-30	-71	-34
	0.046	828	-2	-90	11
			-3	-89	20
			-8	-69	15
	0.004	2540	31	-61	-47
			37	-57	-40
			27	-69	-36
	<0.001	9370	-37	-2	-40
			-58	-6	-21
			-57	-34	-23
	0.015	1541	17	-87	24
			16	-83	40
			9	-85	32
	0.02	1327	8	44	-10
			8	52	-18
			7	59	-12
	0.005	2335	-46	-49	46
			-49	-41	47
			-34	-44	35
	0.02	1343	-17	60	-6
			-20	62	3
			-9	58	-5
	0.046	823	36	-75	28
			40	-80	10
			43	-75	21
**R1 (negative association)**	<0.001	96934	23	19	10
			-21	7	47
			21	32	3
	0.013	1591	39	-67	-35
			37	-56	-37
			21	-77	-41
	0.035	962	-31	-68	-34
			-23	-71	-42
			-38	-51	-39
	<0.001	5947	-16	-75	7
			-34	-82	23
			-19	-82	-4
	0.002	3116	-50	-55	-3
			-47	-64	3
			-44	-46	-2
	0.036	959	-44	-49	46
			-48	-60	41
			-49	-41	47

	0.018	1371	-55	-25	26
			-58	-32	35
			-55	-15	29
**R2* (negative association)**	0.005	1700	28	-54	19
	<0.001	3094	-24	38	11
			-23	31	4
			-30	30	21
	0.033	800	-27	-49	-46
			-28	-51	-35
	0.047	654	-31	-63	5
			-26	-59	17

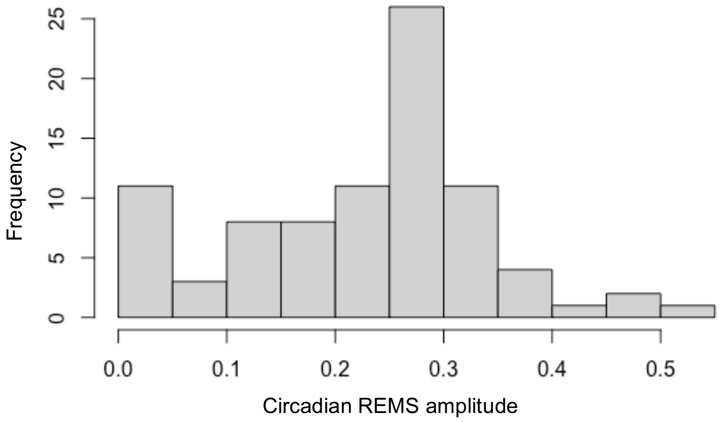

Supplementary Figure 1: Distribution of circadian REMS amplitude values (N=86).

Supplementary Table 4: Statistical VBQ outputs of the association between circadian REMS amplitude and MTsat, R1 or R2* white matter values.

	Cluster p(FWE-corr)	Cluster size (n voxels)	Peak coordinates (mm) x	y	z
**MTsat (positive association)**	0.013	1642	20	12	24
			21	2	28
**R1 (positive association)**	<0.001	10215	-21	-19	28
			-16	-26	30
			-2	-26	18
	0.012	1670	-47	-16	-25
			-41	-17	-13
			-44	-1	-21
	<0.001	5163	17	-4	32
			22	-26	29
			20	-15	37
	0.011	1718	-28	54	-7
			-23	50	2
			-18	55	14
	0.015	1512	31	-60	16
			33	-56	23
			26	-54	28
**R2* (positive association)**	<0.001	3145	24	35	9
			18	24	18
	0.044	680	38	-40	-2
	0.003	1881	-20	-25	31
			-29	-18	36
			-26	-34	41
	0.029	850	-27	-43	21
			-34	-42	4
			-26	-43	29
	0.008	1457	22	-29	29
			19	7	27
			21	-20	31

Supplementary Table 5: Statistical VBQ outputs of the association between circadian REMS amplitude and MTsat, R1 or R2* grey matter values.

	Cluster p(FWE-corr)	Cluster size (n voxels)	Peak coordinates (mm) x	y	z	Brain regions
**R1 (positive association)**	0.001	1546	-19	-16	24	Left Thalamus, Left Caudate, Left Ventral Diencephalon
			-9	-3	9	
			-2	0	-13	
	0.049	521	25	-11	-27	Right Hippocampus, Right Parahippocampal Gyrus
			18	-16	-25	
			34	-10	-26	
