# Supplementary material for: Circadian rapid eye movement sleep expression is associated with brain microstructural integrity in older adults
Source: Commun Biol. 2024 Jun 22;7:758. doi: 10.1038/s42003-024-06415-y (PMC11193799; doi:10.1038/s42003-024-06415-y)
Supplement: Supplementary file 2 — Description of Additional Supplementary Materials [file 42003_2024_6415_MOESM2_ESM.docx]

**Description of Additional Supplementary Files**

**File name:** Supplementary Data 1

**Description:** Raw data used for Figure 2
