# Supplementary material for: Circadian rapid eye movement sleep expression is associated with brain microstructural integrity in older adults
Source: Commun Biol. 2024 Jun 22;7:758. doi: 10.1038/s42003-024-06415-y (PMC11193799; doi:10.1038/s42003-024-06415-y)
Supplement: Supplementary file 4 — Reporting Summary [file 42003_2024_6415_MOESM4_ESM.pdf]

nature portfolio

Corresponding author(s): Marion Baillet

Last updated by author(s): May 6, 2024

Reporting Summary

Nature Portfolio wishes to improve the reproducibility of the work that we publish. This form provides structure for consistency and transparency in reporting. For further information on Nature Portfolio policies, see our Editorial Policies and the Editorial Policy Checklist.

Statistics

For all statistical analyses, confirm that the following items are present in the figure legend, table legend, main text, or Methods section.

n/a	Confirmed	
☐	☒	The exact sample size (*n*) for each experimental group/condition, given as a discrete number and unit of measurement
☐	☒	A statement on whether measurements were taken from distinct samples or whether the same sample was measured repeatedly
☐	☒	The statistical test(s) used AND whether they are one- or two-sided<br>*Only common tests should be described solely by name; describe more complex techniques in the Methods section.*
☐	☒	A description of all covariates tested
☐	☒	A description of any assumptions or corrections, such as tests of normality and adjustment for multiple comparisons
☐	☒	A full description of the statistical parameters including central tendency (e.g. means) or other basic estimates (e.g. regression coefficient) AND variation (e.g. standard deviation) or associated estimates of uncertainty (e.g. confidence intervals)
☐	☒	For null hypothesis testing, the test statistic (e.g. *F*, *t*, *r*) with confidence intervals, effect sizes, degrees of freedom and *P* value noted<br>*Give P values as exact values whenever suitable.*
☒	☐	For Bayesian analysis, information on the choice of priors and Markov chain Monte Carlo settings
☐	☒	For hierarchical and complex designs, identification of the appropriate level for tests and full reporting of outcomes
☐	☒	Estimates of effect sizes (e.g. Cohen's *d*, Pearson's *r*), indicating how they were calculated

Our web collection on statistics for biologists contains articles on many of the points above.

Software and code

Policy information about availability of computer code

Data collection	- Sleep-EEG data was acquired using RemLogic software (EMBLA, Natus Medical Incorporated, Planegg, Germany).<br>- Saliva samples were obtained by passive drooling.<br>- MRI data was collected using a 3T scanner (MAGNETOM Prisma, Siemens) equipped with a 64-channel head coil.
Data analysis	- Sleep stages were scored in 30-second epochs according to the American Academy of Sleep Medicine criteria using a previously validated automatic sleep scoring algorithm (ASEEGA, PHYSIP, Paris, France)<br>- Salivary melatonin levels were analysed via liquid chromatography coupled to a tandem mass spectrometer. Secretion profiles were determined with a skewed baseline cosine function.<br>- MRI data was processed and analyzed using the hMRI toolbox implemented in SPM12.<br>- All statistical analyses were carried out in R and SPM12.

For manuscripts utilizing custom algorithms or software that are central to the research but not yet described in published literature, software must be made available to editors and reviewers. We strongly encourage code deposition in a community repository (e.g. GitHub). See the Nature Portfolio guidelines for submitting code & software for further information.

Data

Policy information about availability of data

All manuscripts must include a data availability statement. This statement should provide the following information, where applicable:

- Accession codes, unique identifiers, or web links for publicly available datasets
- A description of any restrictions on data availability
- For clinical datasets or third party data, please ensure that the statement adheres to our policy

All data and materials supporting this paper will be openly available upon publication at the following repository: 10.5281/zenodo.8434929.

Research involving human participants, their data, or biological material

Policy information about studies with human participants or human data. See also policy information about sex, gender (identity/presentation), and sexual orientation and race, ethnicity and racism.

Reporting on sex and gender	The study sample included 32 females (37%) and 54 (63%) males. Biological sex was determined using questionnaire at enrollement and was used as covariate in statistical analysis. As sex difference in sleep-wake phenotype and brain microstructural inetgrity was not the main scope of this study, no sex interaction was investigated in the analysis.
Reporting on race, ethnicity, or other socially relevant groupings	All participants included in the study were Caucasian.
Population characteristics	The mean age ± standard deviation of the study sample was 68.9 ± 5.2 years, and 32 participants were females. All participants included were cognitively unimpaired (education 14.5 ± 3.2 years; Mini-mental state examination score 29.3 ± 0.8).
Recruitment	Recruitment was performed through access to a GDPR-compliant database in the laboratory and via study advertisement in newspapers. Participants were recruited according to their napping habits, which was subjectively assessed through a questionnaire. Prospective nap recruitment can be considered as a tool to enhance inter-individual variation in sleep-wake phenotypes and more particularly in the temporal dynamics of sleep-wake regulation.
Ethics oversight	The study was approved by the local Ethics Committee of the University Hospital and of the Faculty of Psychology, Logopedics and Educational Sciences at the University of Liège (Belgium) and was conducted in accordance with the Declaration of Helsinki.

Note that full information on the approval of the study protocol must also be provided in the manuscript.

Field-specific reporting

Please select the one below that is the best fit for your research. If you are not sure, read the appropriate sections before making your selection.

☒ Life sciences ☐ Behavioural & social sciences ☐ Ecological, evolutionary & environmental sciences

For a reference copy of the document with all sections, see nature.com/documents/nr-reporting-summary-flat.pdf

Life sciences study design

All studies must disclose on these points even when the disclosure is negative.

Sample size	Based on a study from Oosterman et al. 2008 linking rest-activity rhythms amplitude and white matter integrity with an effect size of f=0.25, power calculation demonstrated that a sample size of n>45 will be sufficient to achieve a statistical power of 0.90 (two-sided alpha = 0.05) in regression models with 8 predictors.
Data exclusions	No data was excluded from analysis.
Replication	Our findings linking circadian REM amplitude and brain microstructural integrity are new and based on a unique experimental design including quantitative multiparameter mapping imaging and a 40-h multiple nap protocol. We are therefore not able to reproduce these findings in independent samples. Yet, our results are similar to previous findings from the literature:<br>- Circadian sleep propensity and circadian REM sleep propensity (Wurts et al. 2000, Dijk et al. 1995, Haimov et al 1997, Münch et al. 2005).<br>- Association between age and brain microstructural integrity estimated using quantitative multiparameter mapping imaging (Callaghan et al. 2014, Draganski et al. 2011).
Randomization	These participants were part of a broader interventional and longitudinal study aiming at investigating the predictive value of circadian regulation on age-related cognitive decline and brain changes. In this context, participants were divided into two groups according to their self-reported napping habits and then underwent an interventional protocol during one year after the baseline visit. This study used however MRI and sleep-EEG data collected during the baseline visit which was equivalent for both groups.

Blinding	The investigators were not blinded to group allocation during data collection. Indeed, sleep schedules (i.e. sleep and wake-up times from baseline night and subsequent nap timing) during the in-lab sleep protocol were individually adapted to the participants' preferred sleep–wake times derived from 7 days of actigraphy before laboratory entrance. However, investigators were blinded to group allocation during statistical analysis.

Reporting for specific materials, systems and methods

We require information from authors about some types of materials, experimental systems and methods used in many studies. Here, indicate whether each material, system or method listed is relevant to your study. If you are not sure if a list item applies to your research, read the appropriate section before selecting a response.

Materials & experimental systems

n/a	Involved in the study	
☒	☐	Antibodies
☒	☐	Eukaryotic cell lines
☒	☐	Palaeontology and archaeology
☒	☐	Animals and other organisms
☒	☐	Clinical data
☒	☐	Dual use research of concern
☒	☐	Plants

Methods

n/a	Involved in the study	
☒	☐	ChIP-seq
☒	☐	Flow cytometry
☐	☒	MRI-based neuroimaging

Plants

Seed stocks	*Report on the source of all seed stocks or other plant material used. If applicable, state the seed stock centre and catalogue number. If plant specimens were collected from the field, describe the collection location, date and sampling procedures.*
Novel plant genotypes	*Describe the methods by which all novel plant genotypes were produced. This includes those generated by transgenic approaches, gene editing, chemical/radiation-based mutagenesis and hybridization. For transgenic lines, describe the transformation method, the number of independent lines analyzed and the generation upon which experiments were performed. For gene-edited lines, describe the editor used, the endogenous sequence targeted for editing, the targeting guide RNA sequence (if applicable) and how the editor was applied.*
Authentication	*Describe any authentication procedures for each seed stock used or novel genotype generated. Describe any experiments used to assess the effect of a mutation and, where applicable, how potential secondary effects (e.g. second site T-DNA insertions, mosiacism, off-target gene editing) were examined.*

Magnetic resonance imaging

Experimental design

Design type	Structural MRI - quantitative multiparameter mapping protocol (MPM)
Design specifications	Acquisition time for the MPM protocol: 30 minutes
Behavioral performance measures	No behavioral performances were measured during the MRI session

Acquisition

Imaging type(s)	Structural MRI
Field strength	3T
Sequence & imaging parameters	The MPM protocol was composed of three co-localized multi-echo 3D FLASH scans 53 acquired with predominant T1, proton density (PD) and magnetization transfer (MT) weighted with TR/FA = 24.5 ms/21° for T1 weighted (T1w), TR/FA=24.5 ms/6° for MT weighted (MTw) and PD weighted (PDw) and a voxel size of 1x1x1 mm3. One radio frequency (RF) transmit field map and one B0 field map were also acquired to correct for B1 field bias and image distortion, respectively. To provide MTw, an off-resonance Gaussian RF pulse of 5 ms duration, 220° nominal flip angle, 2 kHz frequency offset was performed prior to the excitation. Two unaccelerated, low resolution (8 mm isotropic) volumes were acquired prior to the FLASH scans (TR = 6 ms, TE = 2.20 ms, FA = 6°), using respectively the 64-channel coil for signal reception and the body coil for signal reception to correct for the relative receive field sensitivity of the array coil, which is position-specific. B1 maps were extracted from the spin echo (SE) and stimulated echo (STE) signals of a fast 3D multishot echo-planar imaging (EPI) sequence. The multishot EPI data were acquired with the following parameters: matrix size = 64x48x48, FOV = 256x192x192 mm³, TE(SE)/TE(STE)/TM/TR = 25.5/51.0/40.0/500 ms, excitation nominal FA = 90°. The respective flip angles for the SE/STE pulses varied between 130/65° and 280/140° in steps of 15/7.5°.
Area of acquisition	Whole brain scan

Diffusion MRI ☐ Used ☒ Not used

Preprocessing

Preprocessing software	MPM data was processed with the hMRI toolbox (v0.2.2) within the SPM12 environment (revision 12.6)
Normalization	Grey matter and white matter probability maps, derived from the segmentation of the MTsat maps, were used to create a study-specific DARTEL template. MTsat, R1 and R2* maps were then non-linearly and linearly warped to the MNI space using the subject-specific diffeomorphic estimates from the DARTEL procedure and an affine transformation, respectively.
Normalization template	The normalization template was a DARTEL study-specific template.
Noise and artifact removal	During the creation of MPM maps, three pairs of radio frequency sensitivity maps (one pair per contrast: T1w, PDw and MTw) were used to correct for the relative receive field sensitivity of the array coil, which is position-specific. In addition, one radio frequency (RF) transmit field map and one B0 field map were also used to correct for B1 field bias and image distortion, respectively.
Volume censoring	No volume censoring was used.

Statistical modeling & inference

Model type and settings	Whole-brain voxel-based quantification analyses were performed in the framework of SPM12 using multiple linear regression models.
Effect(s) tested	Main effect of circadian REM sleep amplitude on quantitative MPM maps.

Specify type of analysis: ☒ Whole brain ☐ ROI-based ☐ Both

Statistic type for inference (See Eklund et al. 2016)	Inferences were performed using whole-brain family-wise error (FWE) corrected p-values with a threshold set at $p < 0.05$, at the cluster level (cluster forming threshold at $p < 0.0001$ uncorrected at the voxel level to favor parsimony in the construction of the model).
Correction	Family-wise error (FWE) correction at the cluster level.

Models & analysis

n/a	Involved in the study
☒	☐ Functional and/or effective connectivity
☒	☐ Graph analysis
☒	☐ Multivariate modeling or predictive analysis
